# Predicting gene regulatory interactions based on spatial gene expression data and deep learning

**DOI:** 10.1371/journal.pcbi.1007324

**Published:** 2019-09-17

**Authors:** Yang Yang, Qingwei Fang, Hong-Bin Shen

**Affiliations:** 1 Center for Brain-Like Computing and Machine Intelligence, Department of Computer Science and Engineering, Shanghai Jiao Tong University, Shanghai, China; 2 Key Laboratory of Shanghai Education Commission for Intelligent Interaction and Cognitive Engineering, Shanghai, China; 3 School of Bio-medical Engineering, Shanghai Jiao Tong University, Shanghai, China; 4 Institute of Image Processing and Pattern Recognition, and Key Laboratory of System Control and Information Processing, Ministry of Education of China, Shanghai Jiao Tong University, Shanghai, China; Yale University, UNITED STATES

## Abstract

Reverse engineering of gene regulatory networks (GRNs) is a central task in systems biology. Most of the existing methods for GRN inference rely on gene co-expression analysis or TF-target binding information, where the determination of co-expression is often unreliable merely based on gene expression levels, and the TF-target binding data from high-throughput experiments may be noisy, leading to a high ratio of false links and missed links, especially for large-scale networks. In recent years, the microscopy images recording spatial gene expression have become a new resource in GRN reconstruction, as the spatial and temporal expression patterns contain much abundant gene interaction information. Till now, the spatial expression resources have been largely underexploited, and only a few traditional image processing methods have been employed in the image-based GRN reconstruction. Moreover, co-expression analysis using conventional measurements based on image similarity may be inaccurate, because it is the local-pattern consistency rather than global-image-similarity that determines gene-gene interactions. Here we present GripDL (Gene regulatory interaction prediction via Deep Learning), which incorporates high-confidence TF-gene regulation knowledge from previous studies, and constructs GRNs for *Drosophila* eye development based on *Drosophila* embryonic gene expression images. Benefitting from the powerful representation ability of deep neural networks and the supervision information of known interactions, the new method outperforms traditional methods with a large margin and reveals new intriguing knowledge about *Drosophila* eye development.

## Author summary

Gene expression images, with abundant spatial expression patterns, have become an important resource for identifying gene regulatory networks (GRNs), while the computational methods for image-based GRN reconstruction have been very few. In spite of the difference in experimental types and conditions, we utilize previously verified TF-gene interactions by RNA-Seq data and motif analysis as training labels, and design a supervised deep learning method, GripDL, for the prediction of GRNs using gene expression images. We demonstrate its performance by inferring large-scale GRNs for Drosophila eye development based on spatial expression patterns of *Drosophila* embryos. The GRNs constructed by GripDL not only show high consistency with previous work, but also reveal important regulators in the early stage of *Drosophila* eye formation.

## Introduction

Over the past decades, the advances of high-throughput technologies have led to a rapid accumulation of genomic, transcriptomic, proteomic and metabolomics data, and enabled the studies of gene regulation and gene-gene interactions at genome scale [1, 2]. Especially, the reconstruction of gene regulatory network (GRN) has been a hot topic in the field of bioinformatics [3]. GRNs are usually represented by a graph data structure, where nodes and edges denote genes and their interactions, respectively. Given a gene set, the reverse engineering algorithms for GRNs aim to identify edges between nodes so as to infer the network structure, where the edges (interactions) have two major types, i) physical/direct interactions, i.e., interactions between transcription factors (TFs) and their target genes, usually revealed by ChIP-Chip or ChIP-Seq experiments; ii) influential/indirect interactions (i.e. gene interaction network, GIN), inferred by similar expression levels from DNA microarray or next-generation sequencing profiles [4]. The identification of both types has attracted a lot of research interests [5], though there may be not a clear distinction between GRN and GIN.

**Fig 1:**
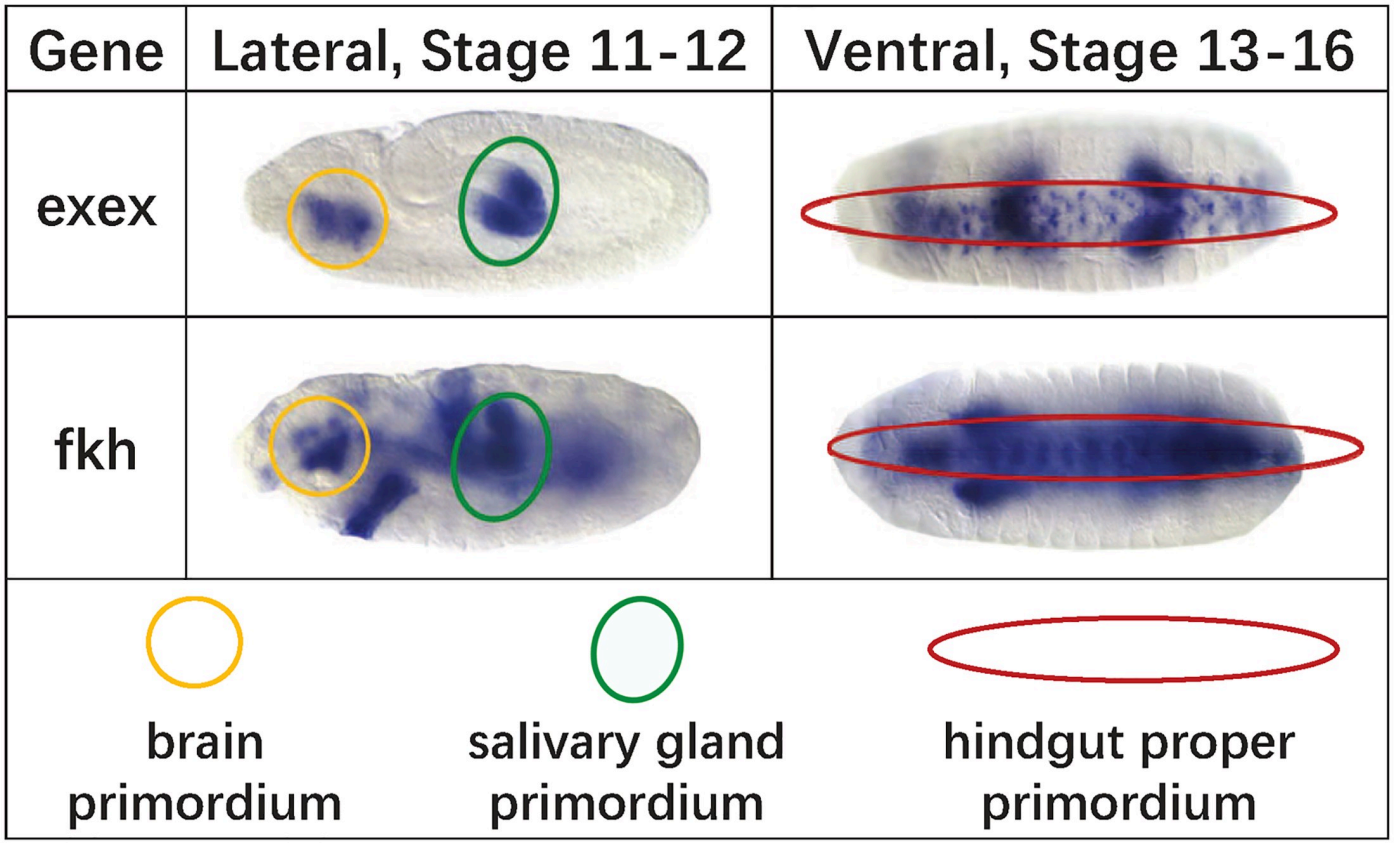
Examples of gene expression images. Two genes, *exex* and *fkh*, and their ISH images of lateral and ventral directions. Although the two genes are not similar from the global view, they have three annotation terms in common, i.e. brain primordium, salivary gland primordium and hindgut proper promordium, whose corresponding expression regions are marked by yellow, green, and red circles, respectively.

For the past decades, a lot of models have been developed for the reconstruction of gene regulatory networks. The major types of models include linear regression [6], mutual information [7, 8], Pearson’s/Spearman’s correlation [8], Bayesian networks [9], etc. The GRN inference is a notoriously challenging task. According to the DREAM (reverse engineering assessment and methods) project [10], which holds contests for GRN inference, no single method performs the best across all data sets. Marbach et al. proposed a community network combining the predictions of all 35 participating teams and achieved the best results [10], but its precision on the high-confidence network of *E. coli* and *S. aureus* is only around 50%. A major reason is that these methods work on scalars from high-throughput experimental data, e.g. the gene expression levels from microarrays, and they identify gene regulation relationships based on the similarity or correlation of expression levels. Since the scalars are averaged values across the tissue or whole body, the similarity based on expression levels may not truly reflect the association between two genes. For example, Fig 1 shows some images of two genes, *exex* and *fkh*, which have three annotation terms in common. Their spatial expression patterns are very similar at local regions; while comparing at the whole embryo scale, the expression level of *fkh* is much higher than that of *exex*, thus their regulation relationship may be missed by traditional inference models. Puniyani et al. also provided an example (Fig 1 in [11]), in which two genes have completely different spatial expression patterns over time, but their averaged values are nearly identical, suggesting that the averaging operation would lead to unreliable results.

Thanks to the abundant spatial expression data, gene expression images have become a new resource for GRN inference [12], and image-driven methods for constructing GRNs are emerging [13, 14]. Although the mining of spatial expression patterns has resulted in many new findings, the image-based GRN reconstruction has still been highly underdeveloped. A major problem is that many existing studies of image-based GRN/GIN inference rely on measuring the similarity between images, with the assumption that similar images suggest a co-expression relationship. For instance, for the *Drosophila* gene expression images, Puniyani and Xing [13] proposed a method called GINI based on a multi-variate Gaussian model to build the gene interaction network. They used a high-dimensional feature vector to represent a gene, where each feature denotes the expression magnitude in a spatial location of the embryo. However, due to the complexity of biological system, gene interaction patterns are usually present in local regions of images or in special forms of association between image features, thus methods based on image similarity may not fit in this task. As shown in Fig 1, the local similarity holds great significance for investigating spatial expression patterns. Wu *et al*. noticed the local patterns, and adopted a non-negative matrix factorization (NMF) method to encode the *Drosophila* embryonic images into principal patterns. Based on the sparse representation, they built spatially local networks [14]. Nevertheless, the unsupervised methods, due to the nature of lacking supervision information, have inherent shortcomings in accurately figuring out the gene regulatory relationships.

Moreover, till now, only traditional image feature extraction methods have been applied in the image-based GRN reconstruction, such as scale invariant feature transform (SIFT) [15] and sparse coding [16–18]; while the state-of-the-art deep learning models have not been employed yet.

Considering the contrast between underdeveloped computational tools in identifying GRNs based on images and the ever-expanding spatial expression data, new protocols are in great demand. In this study, we propose a new method for the prediction of gene regulatory interactions, named GripDL, which is a supervised deep model, driven by already-known TF-target gene interaction knowledge. In other words, it discards the image-similarity assumption but learns autonomously from the known data what kind of features determine the interaction between genes.

We assess the performance of GripDL via a large-scale GRN for *Drosophila* eye development [19]. As a model organism, *Drosophila* has been extensively studied for understanding the development mechanisms of animals. Especially, the formation of visual system and retina differentiation have attracted a lot of research interests. Much efforts have been put on the inference of GRNs for *Drosophila* eye development, including the studies using microarray, RNA-Seq, Chip-Seq and sequence analysis [19, 20]. However, large-scale analysis based on spatial gene expression data has been lacked. We utilize the spatial gene expression data generated by *in situ* hybridization (ISH) imaging technology, provided by the Berkeley *Drosophila* Genome Project (BDGP) (www.fruitfly.org) [21, 22]. The current release (Oct. 2018) includes over 130,000 ISH images from 8390 genes captured at different developmental stages of *Drosophila* embryogenesis. Although the spatial gene expression data is obtained from embryos, our experimental results demonstrate its usefulness in the identification of the GRNs for eye development, especially for uncovering the regulators functioning in the initial stages for establishing the visual system. We extract the ground truth knowledge from a known GRN of *Drosophila* eye development revealed by RNA-Seq experiments and motif prediction [19]. The experimental results show that the supervised deep learning model significantly outperforms the existing reconstructing algorithms based on the same image resource, and it reveals important transcript factors whose regulatory roles have not been fully recognized yet.

## Materials and methods

### Data preparation

#### Spatial expression database

We extract the pre-processed ISH images from FlyExpress (http://www.flyexpress.net) [23–25]. In this database, the raw ISH images from BDGP database have been cropped, aligned, and scaled to the size of 320 × 128. As in the BDGP database, the standardized images are assigned to 16 embryonic stages, each gene corresponds to a group of images, and a set of CV (controlled vocabulary) terms.

#### GRN for *Drosophila* eye development

The ground truth TF-target gene interactions, i.e. the verified interactions, are from the study of Ref. [19], in which a large-scale gene regulatory network on *Drosophila* eye development was established. We regard it as valid because they considered both co-expression relationships (by RNA-Seq) and physical interactions (using computational motif inference) to yield the TF-target associations. Moreover, the authors marked confidence levels for the GRNs, namely, high-, medium and low-confidence, and they released the high- and medium- GRNs. In the high-confidence GRN, TF-target links were drawn from direct evidence, while the medium-confidence network contains the links with partial evidence.

#### Construction of the data sets

Note that the organism samples used in Potier’s study [19] are fruit fly larvae, because the eye development mainly happens during the larval stage [26]. In fact, the eye development already begins early in embryo (a lot of genes are annotated by eye-related terms in BDGP), and BDGP collects only embryonic images, thus we use the images from the last stage range of embryonic period, i.e. stage 13-16. In order to build a benchmark set, we retrieve the common genes shared between the high-confidence GRN and the last embryonic stage range in FlyExpress, including 96 TFs, 1261 target genes and 2889 TF-gene links. The negative data is randomly selected TF-gene pairs from the same gene set, and the negative pairs are not present in the high- or medium-confidence network. The positive to negative ratio is set to 1:1. We divide the TF-gene pairs into training and test sets, where images belonging to the same gene are either in the training set or in the test set. The training to test ratio is 4:1, and 10% training data is used for validation. In order to prepare an independent test set, we filter out the links common to the high-confidence and medium-confidence networks, leaving the links specific to the medium-confidence network. The statistics of the benchmark dataset and independent test set are shown in Table 1.

**Table 1:** Statistics of the data sets.

	Benchmark data set		Independent test set	
TF #	96		128	
Target gene #	1261		2096	
	Positive	Negative	Positive	Negative
TF-target link #	2889	2889	16459	/
Image pair #	45345	43355	293319	/

### Problem modeling

In this study, we try to determine whether a certain TF regulates a certain gene’s expression according to their ISH images, thus the input is a combination of two image features and output is a probability of the existence of regulating relationship. However, this is not a conventional image classification problem, as each gene corresponds to a set of images, captured in different orientations, i.e. lateral, ventral and dorsal, or from different experimental batches, and the size of set is not fixed. Therefore, in order to employ the state-of-the-art deep learning models, we generate a set of instances for each gene pair, which includes all the cross-gene image pairs, and each pair of images should have the same orientation. Specifically, for a TF *g*_*i*_ and a gene *g*_*j*_, they correspond to two image sets, *X*_*i*_ and *X*_*j*_, respectively. Let *X*_*i*_ be the union of three sets, *X*_*i*,*l*_, *X*_*i*,*v*_, *X*_*i*,*d*_, which contain images of lateral, ventral and dorsal orientation, respectively. And *X*_*j*_ is defined in the same way.

Let Y be the output space, and *y*_*i*,*j*_(∈ {0, 1}) be the output label, indicating whether the interaction between *g*_*i*_ and *g*_*j*_ exists or not. In the original learning scenario, we want to learn a mapping function *f* as shown in Eq (1),

$$\begin{array}{c} y_{i,j}=f\left(X_{i},X_{j}\right), \end{array}$$ (1)

where the input consists of two varying sized image sets. To simplify this multi-instance learning problem, we split the pair (*X*_*i*_, *X*_*j*_) into multiple pairs of single images, e.g. $\left\{x_{i}^{\left(p\right)},x_{j}^{\left(q\right)}\right\}$, where $x_{i}^{\left(p\right)}$ is the *p*th image in *X*_*i*_, $x_{j}^{\left(q\right)}$ is the *q*th image in *X*_*j*_, and $x_{i}^{\left(p\right)}$ and $x_{j}^{\left(q\right)}$ have the same orientation. In the training phase, we assign the same label *y*_*i*,*j*_ to all the pairs splitted from (*X*_*i*_, *X*_*j*_), and we try to learn a mapping function *f*′, which satisfies Eq (2),

$$\begin{array}{c} y_{i,j}=f^{\prime }\left(x_{i}^{\left(p\right)}⊕x_{j}^{\left(q\right)}\right), \end{array}$$ (2)

where the ⊕ operator concatenates the two vectors into a whole feature vector, then the task is converted into a single-instance learning problem in conventional supervised learning scenario. Note that a single image may not cover all the representative expression patterns of its corresponding gene, thus the above simplification may cause some problem, but according to the previous studies, the single-instance learning works well for the automatic annotation of *Drosphila* embryonic images [17, 27], and another advantage of the conversion to single-instance learning is that it substantially expands the data set.

After training, we obtain the estimated mapping function $\hat{f^{\prime }}$ for prediction. The model outputs a probability value for each pair of images with the same orientation. Since our goal is to predict the regulatory relationship for TF-gene pairs, in the test phase, we need to integrate the outputs of image pairs to the final probability of the TF-target linkage, as shown in Eq (3),

$$\begin{array}{c} {\hat{y}}_{i,j}=\frac{\sum_{o\in \{l,v,d\}}\sum_{p}\sum_{q}\hat{f^{\prime }}\left(x_{i,o}^{\left(p\right)}+x_{j,o}^{\left(q\right)}\right)}{\sum_{o\in \{l,v,d\}}|X_{i,o}\left|\times \right|X_{j,o}|}, \end{array}$$ (3)

where |⋅| denotes the size of a set. We set the threshold to the default value 0.5, i.e., an output probability greater than or equal to 0.5 indicates the existence of regulatory relationship.

### Model architecture

**Fig 2:**
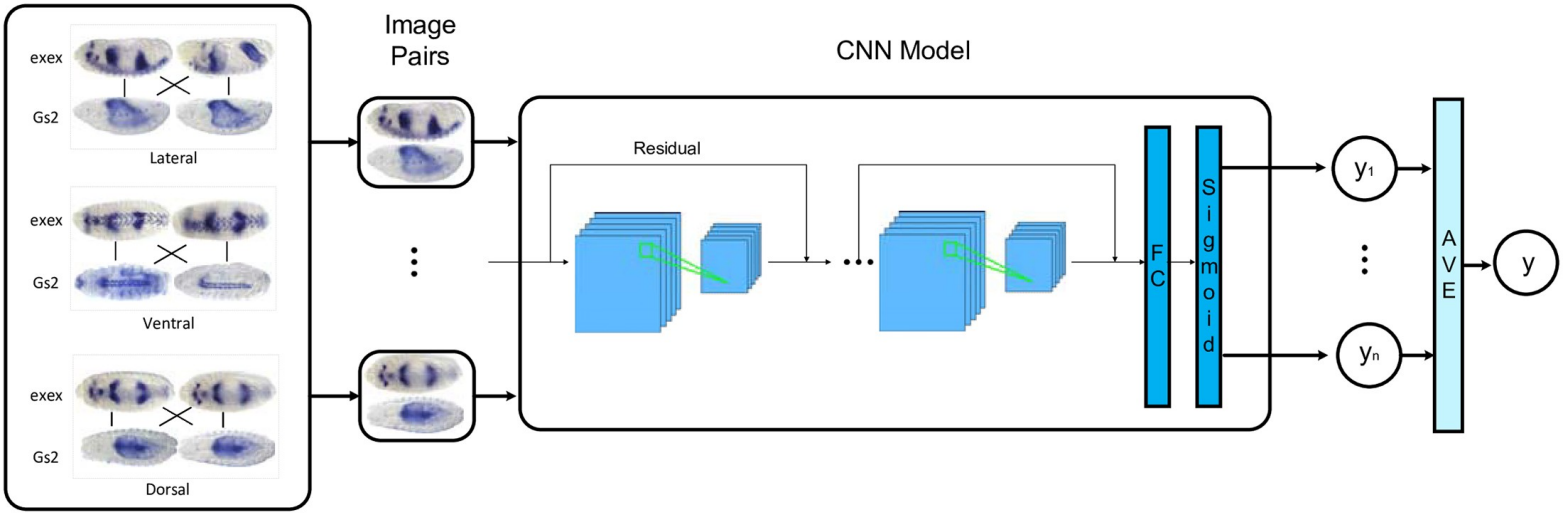
Flowchart of GripDL. Take the TF-gene pair (*exex*, *Gs2*) as an example. The inputs of GripDL are pairs of images. Each pair consists of two images of the same orientation, one is from *exex*, and the other is from *Gs2*. All the outputs of the image pairs are averaged to get the final output.

We model the prediction of gene regulatory interaction as a binary classification problem, in which a data instance corresponds to a gene pair, and a label (positive or negative) denotes the presence or absence of regulatory interaction between the two genes. The data features are extracted from gene expression images. The training labels are from previously revealed GRNs by using RNA-Seq data and computational motif inference [19]. Fig 2 shows the flowchart of GripDL. The convolutional neural network (CNN) serves as a binary classifier. Especially, we adapt ResNet50 [28] model in our prediction system. The top layer of ResNet50 model is replaced by a fully connected layer activated by tanh function with an output dimensionality of 128, where both the batch normalization and dropout (dropout rate 0.1) are used. The 128-D output is fed into the final fully connected layer and gives rise to the prediction probability via a sigmoid activation function. The detailed settings of model architecture is shown in Table 2. There are four sets of residual blocks, namely conv2_x, conv3_x, conv4_x, and conv5_x, which contain different numbers of basic residual units.

**Table 2:** Model architecture of GripDL^a^.

Layer name	Layer settings	Output size
conv1	7×7, 64, stride 2	128×160
conv2_x	3×3 max pooling, stride 2	64×80
	$[\begin{array}{c} 1\times 1,64\\ 3\times 3,64\\ 1\times 1,256 \end{array}]\times 3$	
conv3_x	$[\begin{array}{c} 1\times 1,128\\ 3\times 3,128\\ 1\times 1,512 \end{array}]\times 4$	32×40
conv4_x	$[\begin{array}{c} 1\times 1,256\\ 3\times 3,256\\ 1\times 1,1024 \end{array}]\times 6$	16×20
conv5_x	$[\begin{array}{c} 1\times 1,512\\ 3\times 3,512\\ 1\times 1,2048 \end{array}]\times 3$	8×10
	global avg pooling	1×1
	fc, sigmoid	

^a^ Numbers in the brackets describe the basic residual units of the network. conv2_x, conv3_x, conv4_x, and conv5_x contain 3, 4, 6 and 3 basic units, respectively. The output size denotes the size of the output feature maps.

## Results

### Experimental settings

In order to assess the performance of our method, we conduct experiments on both the benchmark data set and independent test set. On the benchmark data set, we randomly select 80% of the TF-gene pairs for training and validation and the remaining for test. Note that the data partition is at gene-level rather than image-level while the ratio of training image pairs is also close to 80%. Among the 80% data, 90% is used for training and 10% for validation. Furthermore, we predict regulatory interactions between TFs and target genes on the independent test set by using the trained model. As for the evaluation criteria, we adopt common criteria for the assessment on the benchmark test set, including total accuracy and *F*_1_ measure; while for the independent test set, we only use accuracy because all the samples are positive.

The input of GripDL is a concatenated image pair, where two ISH images are aligned vertically; and the output indicates the probability of regulation relationship, where the threshold is set to the default value 0.5. The learning model is ResNet50 [28], pre-trained on ImageNet [29]. Dropout with a ratio of 0.3 is added after full connected layers. The model is trained for 60 epochs using the SGD optimizer with learning rate 0.001. The source code and data is available at https://github.com/2010511951/GripDL.

### GripDL yields high prediction accuracy on the verified gene regulatory network of *Drosophila* eye development

**Fig 3:**
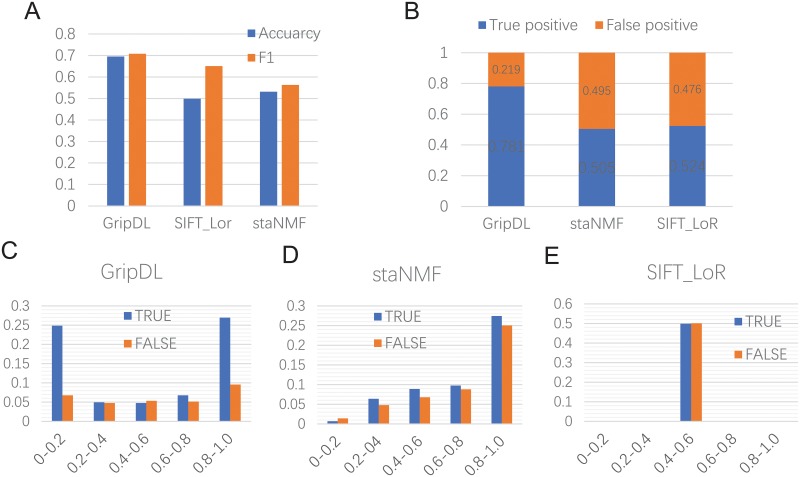
Prediction performance on the benchmark set. (A) shows the overall accuracy and *F*_1_ on the test data in the benchmark set. (B) shows the true and false positives of the top 10% predictions among the test data. (C), (D) and (E) show the distribution of true and false predictions at different output ranges of GripDL, NMF and SIFT_LoR, respectively.

In order to assess the performance of GripDL for predicting gene regulatory interactions, we use a benchmark set derived from a high-confidence GRN of *Drosophila* eye development, where the positive samples are verified TF-gene regulation pairs and negative samples are randomly selected non-regulatory TF-gene pairs. We compare GripDL with two other methods. One is a traditional supervised method, SIFT_LoR, using SIFT feature extraction and logistic regression as the classifier. The other is an unsupervised method, staNMF, i.e. stability-driven nonnegative matrix factorization [14]. As shown in Fig 3(A), GripDL has an obvious advantage over the other two methods. Both the accuracy and *F*_1_ of GripDL are over 14% higher than those of staNMF. Although SIFT_LoR is a supervised method and its *F*_1_ increases about 9% compared to staNMF, its accuracy is only around 50%. (Note that the accuracy reported in this experiment may be underestimated, because unrevealed links may exist in the GRN).

Especially, we focus on the false positive (FP) ratio for the top 10% predictions, whose output probabilities rank top 10% among all test data. According to Fig 3(B), GripDL’s FP ratio is a little above 20%, while the other two methods have nearly 50% FP ratios. Furthermore, we examine the rates of true and false predictions in different output ranges, i.e. [0, 0.2), [0.2, 0.4), [0.4, 0.6), [0.6, 0.8), [0.8, 1.0]. As can be seen in Fig 3(C), GripDL has quite a large differentiation between the positive and negative predictions, as most of the output values concentrate in the first and last ranges. In range [0.8, 1.0], the false positives account for less than 10% of all predictions. In Fig 3(D), most of the NMF predictions fall into the range [0.8, 1.0], and the false positive number is slightly less than the true positive number; while SIFT_LoR performs even worse (Fig 3(E)), whose prediction scores are centered around 0.5, and the FP ratio is also close to 0.5. These results suggest that GripDL captures discriminant features from the image pairs to recognize TF-target links, while neither the unsupervised nor the traditional supervised method is able to provide reliable predictions.

### GripDL validates most of the TF-gene links in the medium-confidence GRN

**Fig 4:**
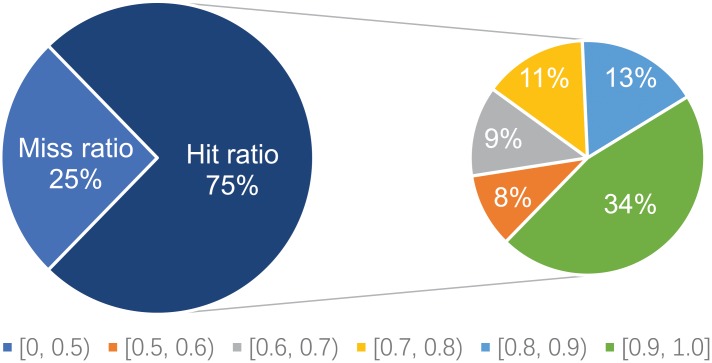
Results on the independent test set. Hit ratio denotes the percentage of links in the set that are identified by GripDL. The right pie presents the percentages of hits with different output ranges.

Besides the benchmark set, we also investigate the consistency between our model predictions and the medium-confidence GRN, i.e. the independent test set, in which the gene interactions lack direct evidence. GripDL identifies around 75% of the links in this set. Again, it has high confidence for most of the positive predictions, as 62.7% of the positive output values are greater than 0.8 and 89.3% are greater than 0.6 (see Fig 4).

Due to the complexity of biological systems, a single type of high-throughput experimental data is often unable to reliably characterize large-scale GRNs, while the regulatory interactions validated by multiple types of experimental data are considered qualified. However, the GRNs inferred by different experimental data often have very small overlap, due to different experimental conditions or the limitations of inference methods. For instance, in Ref. [13], the authors compared their results obtained by the BDGP data with a network inferred by microarray, and found that only 1% of the edges were shared by the 2 networks.

By contrast, in this study, on both the benchmark set and independent test set, the constructed GRNs by GripDL show a high degree of consistency (over 75% common edges) with those reported in Ref. [19]. It can be attributed to the proper supervision information and effective regulatory patterns detected from the images. On the one hand, the training labels have a high quality, as they were validated by both RNA-Seq experiments and motif sequence analysis. On the other hand, it suggests that the gene expression images of the last developmental stage of *Drosophila* embryos indeed contain gene regulatory information for eye development. Guided by high-quality interaction pairs, GripDL learns the regulatory information from the images and predicts unknown interactions. Its predictions are helpful for validation and recognizing new regulatory interactions. A further analysis of the predictions with high probabilities from the medium-confidence GRN is given in the following sections.

### GripDL identifies important regulators in the eye development of *Drosophila*

Although GripDL successfully identifies most of the links in the independent test set, it is still questionable whether the predicted highly probable regulatory interactions are truly biologically meaningful. Unlike the benchmark set, the links in the independent test set lack direct evidence. Thus, we mainly investigate the prediction results for the latter set and seek supporting evidence.

#### Hub gene analysis

**Fig 5:**
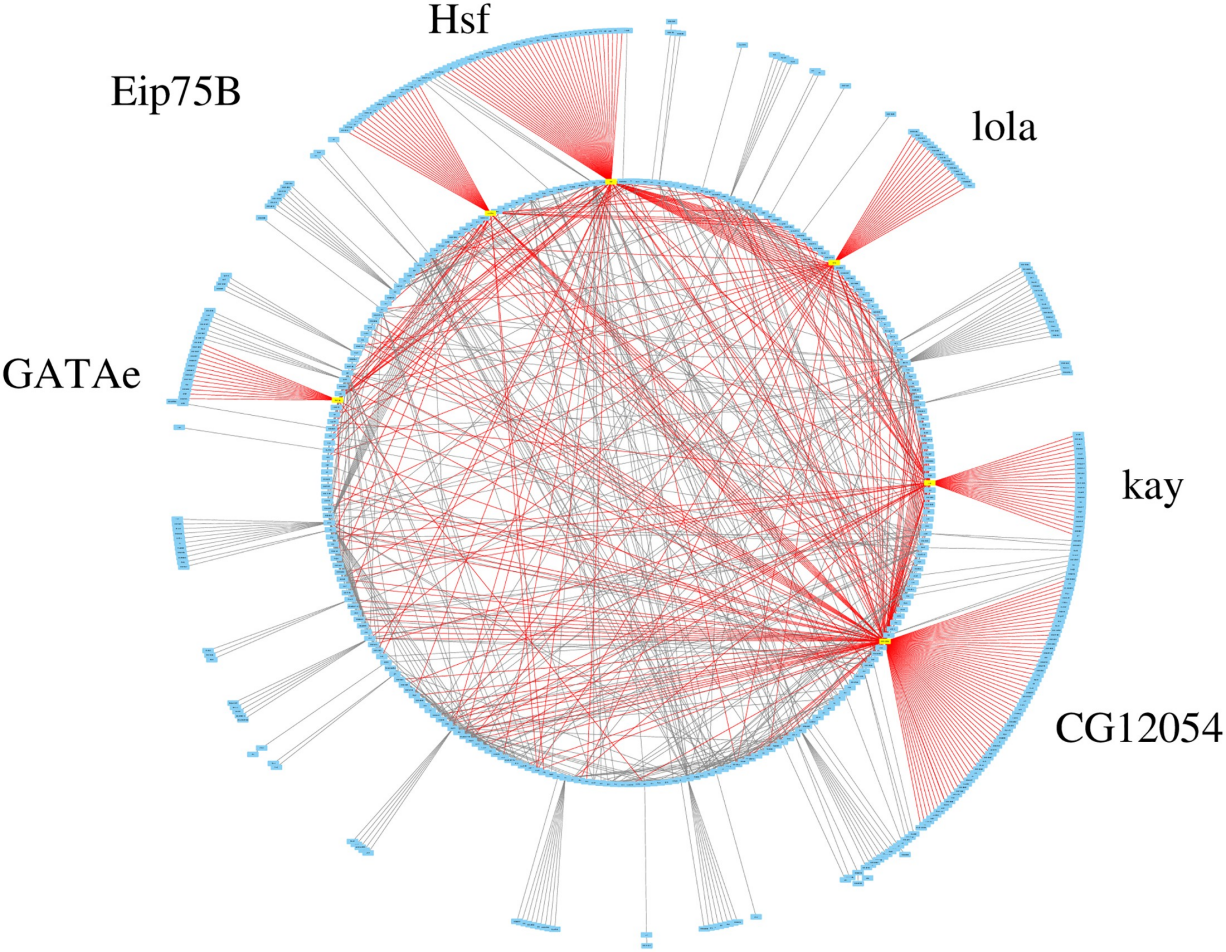
Hub gene analysis. The hub genes are extracted from top 1000 most confident edges.

We first retrieve the hub genes from the prediction results by selecting dominant genes among the top-ranked TF-gene links according to their output probabilities. Fig 5 depicts a circular layout of the top 1000 links/edges (drawn by CytoScape [30]), which clearly reveals 6 hub genes, *CG12054*, *Hsf*, *kay*, *lola*, *Eip75B* and *GATAe*, with 141, 85, 56, 47, 42 and 25 predicted targets, respectively (The target genes are shown in S1 Fig).

Through a literature research, we find some supporting evidence of the association between these hub genes and *Drosophila* eye development. In Ref. [20], Michaut *et al*. conducted two different DNA microarray experiments, where *lola* was detected as expressed in the eye discs with high significance by both arrays. Abrell *et al*. revealed that *Eip75B* is one of the suppressor of the irregular facet mutation [31]. *GATAe* has been identified to be in the kernel of GRN for the development of major body part of animals [32]. Besides, all of these 6 hub TFs are involved in biological processes vital to *Drosophila*’s life cycle, including molecule-level nucleic acid binding, cell-level mitosis, apoptosis, system-level immunity, etc. At molecular level, *kay* is found to be relevant to RNA polymerase II proximal promoter sequence-specific DNA binding [33], and *CG*12054 is responsible for the positive regulation of transcription by RNA polymerase II [34]. At cell level, *kay* regulates the cyclin-dependent protein serine/threonine kinase activity in G2/M transcription of mitotic cell cycle and is associated with the second mitotic wave in compound eye morphogenesis [35], *lola* participates in the nurse cell apoptotic process [36], and *GATAe* is involved in the maintenance of intestinal stem cell homeostasis [37]. At system level, *kay*, *lola*, *Eip75B*, and *GATAe* were identified to play an important role in the regulation of antimicrobial humoral response [38, 39].

As another illustration, we identify the most prominent hub nodes among the top 3000 edges, as shown in S2 Fig. Specifically, the most dominant hub nodes include *l(1)sc*, *grh*, *gt* and *ase*. These hub genes are involved in many basic and essential functional processes of cells and organisms. For example, *l(1)sc* is associated with the regulation of glucose metabolic process [40], *grh* is responsible for the regulation of proliferation in cell mitosis [41], and *gt* is correlated with cell death through phagocytosis [42]. Besides, *ase* and *grh* are both involved in the regulation of protein homodimerization activity [43, 44].

Interestingly, most of these hub genes predicted by GripDL have one common feature that they participate in the nervous system development of *Drosophila*. For example, *gt* guides the generation of axon [45], and *ase*, *grh*, *l(1)sc* are all involved in the regulation of nervous system development [46, 47]. This phenomenon suggests a strong connection between eye development and nervous system. In fact, since optic nerve is a vital structure of eye which enables functional signal transduction from retina to brain cortex, it is reasonable that these genes functioning in nervous system also appear in the regulatory network of eye development.

Another notable finding is that GripDL confirms the role of an important TF, *So*, mentioned in Ref. [19], which is known to participate in eye development of *Drosophila* [48]. There are 128 potential targets of *So* given in the medium confidence network, and 54 of them are assigned with high probabilities (> 0.8) by GripDL, indicating the important role of *So* in the transcriptional control of eye development.

Besides *So*, another TF, *Sob*, draws our attention because of its large number of targets, which was not reported in Ref. [19]. GripDL predicts 77 *sob*-target links with probabilities over 0.8. Actually, Bras-Pereira *et al*. verified its expression in the margin-peripodial cells in early eye discs and critical functions in initializing retinogenesis [49]. It is interesting that the gene’s regulatory role in retinogenesis is uncovered by the spatial gene expression of embryos instead of larvae.

#### Functional enrichment

Besides extracting hub genes, we analyze the gene ontology terms for all genes present in the top 3000 predictions from the independent test set, using DAVID [50] (https://david.ncifcrf.gov/). Table 3 shows the top 25 out of 287 biological process GO terms which are ranked by frequency. The terms, like ‘positive/negative regulation of transcription, DNA-templated’ and ‘regulation of transcription from RNA polymerase II promoter’, suggest the basic gene regulation attributes. Also, basic cell activities, such as ‘regulation of glucose metabolic process’, ‘phagocytosis’ and ‘protein phosphorylation’, are involved in the sub-network. In addition, with respect to the organ and system development of *Drosophila*, the GO term ‘compound eye development’ confirms their roles in *Drosophila*’s eye development process. Again, we find this network is closely related with *Drosophila*’s nervous system, as many nervous system related GO terms, such as ‘neurogenesis’, ‘axon guidance’ and ‘dendrite morphogenesis’, are significantly enriched.

**Table 3:** Enriched GO terms for the top 3000 predictions^a^.

GO term	#	%	p-value
transcription, DNA-templated	81	8.1	3.60e-18
regulation of transcription, DNA-templated	72	7.2	6.20e-12
pos-regulation of transcription from RNA pol II promoter	68	6.8	2.00e-23
neurogenesis	62	6.2	2.50e-03
axon guidance	49	4.9	1.10e-14
oogenesis	45	4.5	4.70e-05
imaginal disc-derived wing morphogenesis	44	4.4	3.30e-09
neg-regulation of transcription from RNA pol II promoter	42	4.2	1.20e-13
regulation of transcription from RNA pol II promoter	40	4.0	3.60e-10
dendrite morphogenesis	37	3.7	8.80e-08
regulation of glucose metabolic process	36	3.6	1.00e-07
lateral inhibition	36	3.6	7.30e-06
pos-regulation of transcription, DNA-templated	32	3.2	5.50e-10
neg-regulation of transcription, DNA-templated	32	3.2	6.80e-10
open tracheal system development	31	3.1	3.90e-11
protein phosphorylation	31	3.1	1.70e-02
neuron projection morphogenesis	30	3.0	2.90e-09
peripheral nervous system development	27	2.7	2.3e-10
ventral cord development	27	2.7	2.30e-10
phagocytosis	27	2.7	7.80e-03
**compound eye development**	26	2.6	7.30e-06
heart development	24	2.4	7.80e-12
**central nervous system development**	23	2.3	1.00e-07
dorsal closure	23	2.3	6.50e-06

^a^ The listed GO terms are all biological process related terms. ‘#’ denotes the number of genes annotated by the GO term, and ‘%’ denotes the percentage of gens annotated by the GO term. ‘pos-regulation’ is short for positive regulation, ‘neg-regulation’ is for ‘negative regulation’ and ‘RNA pol II’ is for ‘RNA polymerase II’.

#### Visualized analysis

**Fig 6:**
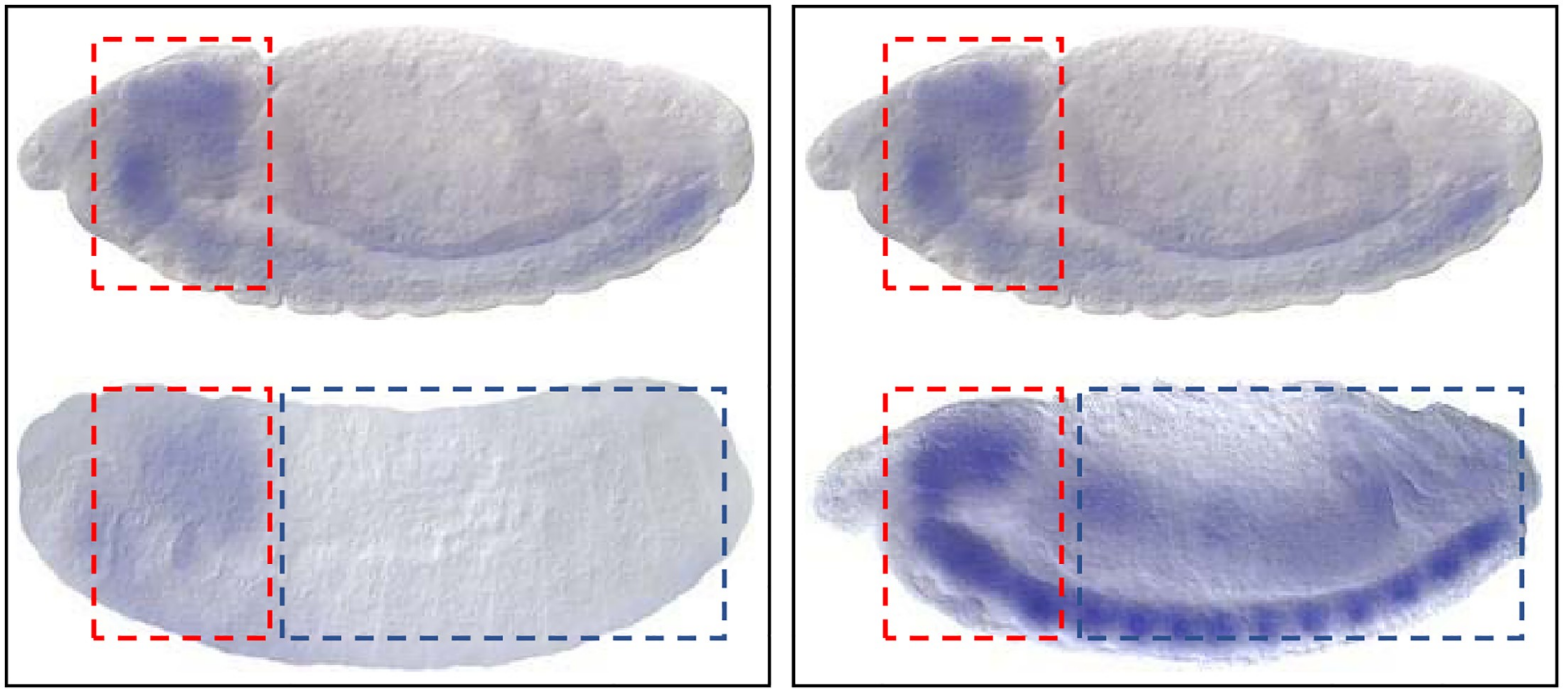
The comparison of expression pattern of *CG12054* and its targets, *lid* and *CG31108*. As arranged in the input, the TF (*CG12054*)’s expression image is put above that of its target, *lid* (left) or *CG31108* (right). Red and blue dotted squares frame the head and body of *Drosophila*’s embryo, respectively.

Since GripDL learns autonomously the expression patterns from image pairs of known TF-target interactions, we explore the hidden patterns via a visualized analysis. Fig 6 presents two image pairs, corresponding to the predicted interactions between the TF *CG12054* and two target genes, *lid* and *CG31108*. As can be seen, the expression of *CG12054* and its targets, *lid* and *CG31108*, have a similar pattern in the head; while the gene expression images of *lid* and *CG31108* look very different, especially in the body part. Both *lid* and *CG31108* receive over 0.9999 predicted probabilities linking to *CG12054*. It demonstrates that it is the local pattern rather than global image similarity that determines gene regulatory interaction.

**Fig 7:**
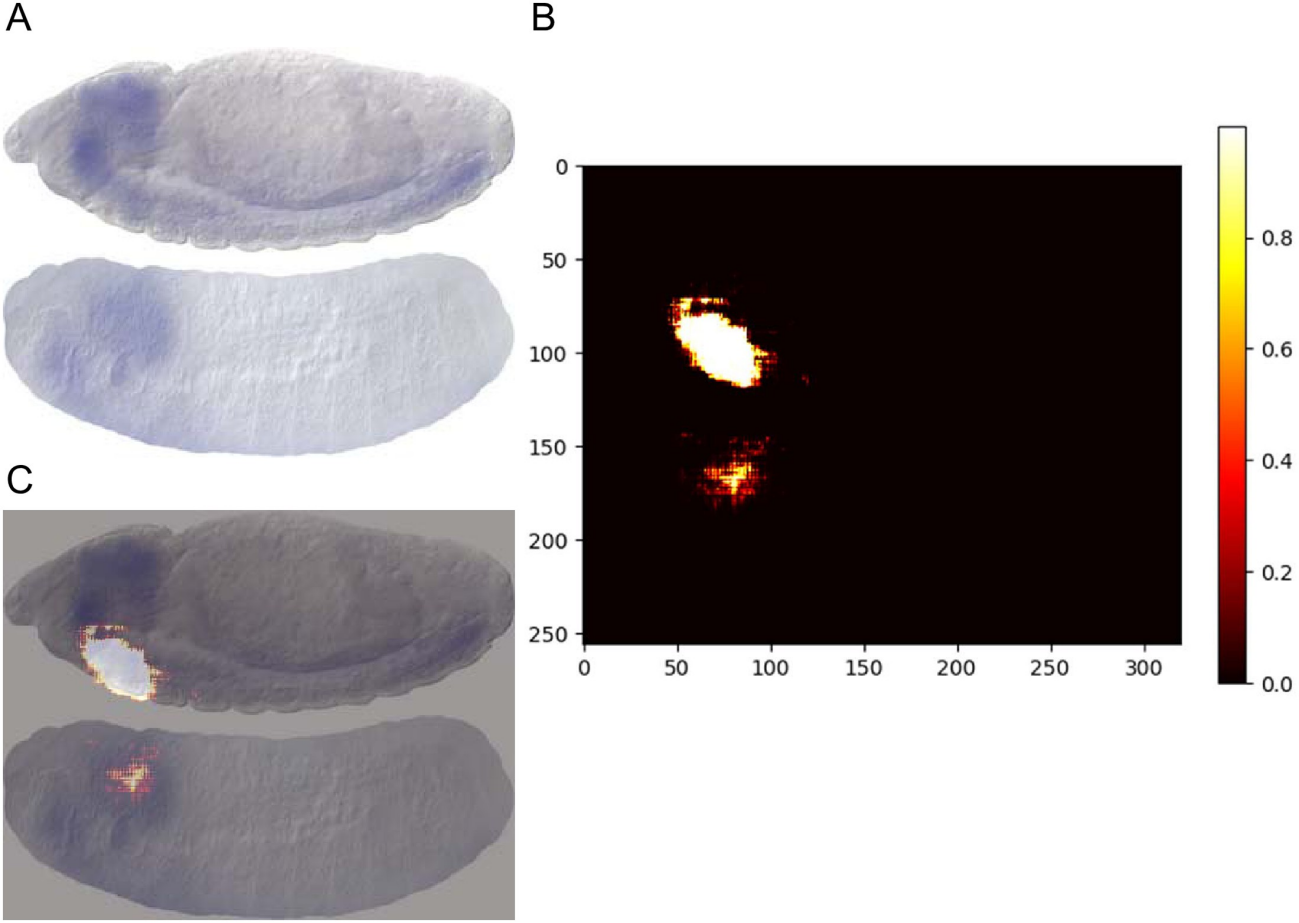
The occlusion test result on the image pair of *CG12054* and *lid*. (A) The original concatenated image of *CG12054* and *lid*. (B) The occlusion map generated by the occlusion test. (C) The merged image of the input and the occlusion map.

Furthermore, we perform an occlusion test to investigate the focus area of GripDL for the prediction. The occlusion test is similar to that used by Kermany et al. [51], which aims to identify the regions that contribute most to the model performance. An occlusion map can be generated by convolving an occluding kernel across the original image. As shown in Fig 7, for the image pair *CG12054* and *lid*, we map the decrease of output probability to pixel intensity in Fig 7(B), i.e. the occlusion map, where the black regions denote no impact on the output probability. It can be observed that there are indeed focus regions of GripDL, and the prediction is mainly affected by the head part of *Drosophila* embryos. This test again demonstrates that GripDL captures the local similarity in spatial expression profiles.

## Discussion

### Supervised methods versus unsupervised methods

By regarding the gene network inference as a machine learning problem based on spatial expression data, both supervised and unsupervised methods could be employed. A major reason for relatively few studies on supervised methods is the lack of large-scale known GRNs. When training samples are scarce, supervised methods have very limited advantages due to the overfitting issue, while unsupervised methods have fewer limitations on the applications. However, unsupervised methods often have pre-defined assumptions about the data, e.g. the spatial independence assumption [13], where the assumption may not hold in the real case. By contrast, supervised methods, especially the deep neural networks, have great capability to learn the complex distributions from supervision information, thus they are more suitable for the inference of a specific functional network with prior knowledge.

From the experimental results, we find that the unsupervised methods can hardly capture the discriminant patterns for the gene regulation in eye development. For one thing, the factors determining gene regulations are not explicitly present in image features, but most likely hidden in the complex temporal-spatial expression associations. For another, supervised learning tends to predict the interactions of the same type as the ground truth, while unsupervised learning may identify other types of interactions. Thus, unsupervised methods tend to predict indirect interactions between two genes other than TF-target interactions, and the predictions may be irrelevant to eye development.

### The impact of image preprocessing

In this study, we use well-curated images from FlyExpress instead of the raw images from BDGP. We investigate the impact of the image preprocessing and conduct a comparison experiment by using the raw images from BDGP. For the high-confidence network of *Drosophila* eye development, we construct a dataset including the same gene pairs (5778 pairs) but much more image pairs (139,620) from BDGP. With the same settings on model architecture and ratios for dividing training, validation and test sets, the BDGP dataset yields 67.6% accuracy and 66.7% *F*_1_ on the test set, which are 1.9% and 4.1% lower respectively, compared with those of FlyExpress dataset. This result suggests the impact of image rotation and translation on the performance of GripDL. Although the data set is greatly augmented, as the raw images contain incomplete or multiple embryos and the embryos in the images are randomly oriented, it is much harder to learn the useful expression patterns from the raw images. Moreover, since we use all images of genes to generate data samples, the low-quality images introduce a lot of noisy samples in the data set, which may hurt the performance.

### The transfer learning strategy

GripDL uses a ResNet pretrained on the ImageNet database [29] as the initial model, which is actually a transfer learning strategy to extract image features. Although biomicroscopy images are very different from natural images, a lot of studies have demonstrated that using CNN models pretrained on ImageNet can obviously enhance the performance on biomedical image processing [52]. It is interesting to examine the performance of original ResNet as a feature extractor for *Drosophila* embryo images, i.e., the weights learned from ImageNet are kept unchanged while only the fully connected layers are adapted to the binary classification problem. In this way, we obtain the accuracy and *F*_1_ on the test set of 0.576 and 0.558, respectively. These results suggest that the gene expression images may share some features with natural images, thus the fixed-weight ResNet still has some discriminant capability, but the fine-tuning is an important step to extract task-specific features.

### Limitations and future works

Note that in the training and test process of GripDL, we need to pick up TFs and their targets beforehand, because the input of GripDL is a pair of aligned images, whose order is fixed, i.e. the TF’s image is located above the target’s image. In the experiments, we find that the order has a big impact on the result. When the image order is opposite between training and test data, the prediction accuracy drops around 10%. This observation indicates that TFs have their distinct spatial expression patterns from normal genes.

When predicting gene regulations, GripDL does not differentiate different modes of regulation, e.g., activation and repression. This is due to the restrictions from the supervision information source, where no activating or repressive information for the large-scale GRN was provided in previous works. As more and more ground truth data become available, where detailed regulatory information can be incorporated into the training process, GripDL can be upgraded to a multi-class predictor to adapt to various kinds of regulation modes.

Another limitation of this study is the data source. Here we adopt a large-scale GRN revealed by RNA-seq and computational motif inference, while there are a lot of GRNs verified by various high-throughput experimental data. For example, Sandmann et al. published a core transcriptional network for early mesoderm development in *Drosophila melanogaster* through chromatin immunoprecipitation followed by microarray analysis (ChIP-on-chip) [53]. Since the mesoderm development occurs during stage 2-4 of embryogenesis, it is very suitable to adopt *Drosophila* embryonic expression images for predicting new regulatory interactions. However, there is only one transcript factor, *Twist*, in the verified network and the data scale is small. As the training of deep neural networks requires sufficient data examples, GripDL may not work well for the inference of small GRNs.

Considering the increasingly enriched experimental data of gene regulations, we will develop methods to integrate multiple sources of data for GRN inference. Moreover, to enhance the generalization ability, we consider to incorporate the image annotations in the GRN reconstruction in the future work. The image annotation task can be used for pretraining, because in BDGP/FlyExpress all the images have manually-curated labels (terms from a controlled vocabulary) and the image annotation task can share the same backbone network with GRN inference. The pretraining strategy may provide a practical way for the reconstruction of GRNs with small data sets. Alternatively, the annotation terms can be regarded as a kind of features and help the inference.

### Conclusion

In recent years, the abundance of spatial expression data has enabled the inference of gene regulatory networks based on spatial distribution of gene expression, and revealed a lot of new regulatory associations that are undetected by traditional experiments. However, for a certain functional gene network, the unsupervised methods which mainly rely on image similarity are incompetent to capture local or hidden patterns associated with the regulatory interaction, and the spatial expression data alone cannot produce reliable results due to noisy data or irrelevant features.

In this study, we incorporate prior knowledge of TF-target regulations into the prediction of unknown regulatory interactions, and design a supervised deep learning model, GripDL, which performs learning and prediction based on spatial expression features. In the experiments on large-scale benchmark data and an independent test set, GripDL achieves significant improvement on the predicting accuracy compared to unsupervised reconstructing methods, suggesting the successful transfer of the TF-target regulation knowledge to the recognition of spatial patterns for identifying new regulatory interactions. And the prediction results not only provide independent evidence for supporting previous high-throughput co-expression analysis but also reveal new biologically meaningful regulatory interactions. This model could also be applied to the inference of gene regulatory interactions for other model organisms, like *Caenorhabditis elegans* and *Danio rerio*, which have some well-studied functional GRNs and gene expression images.

## Supporting information

S1 Fig The hub genes extracted from top 1000 predicted links and their megerd network. The six hub genes, i.e.*CG12054*, *Hsf*, *kay*, *lola*, *Eip75B* and *GATAe*, are highlighted with yellow background.
(TIF)

S2 Fig The hub genes extracted from top 3000 predicted links. (A) shows the targets of *grh* and *l(1)sc*, (B) and (C) show the targets of *gt* and *ase*, respectively.
(TIF)
