# Supplementary figures and images for: Predicting gene regulatory interactions based on spatial gene expression data and deep learning
Source: PLoS Comput Biol. 2019 Sep 17;15(9):e1007324. doi: 10.1371/journal.pcbi.1007324 (PMC6764701; doi:10.1371/journal.pcbi.1007324)

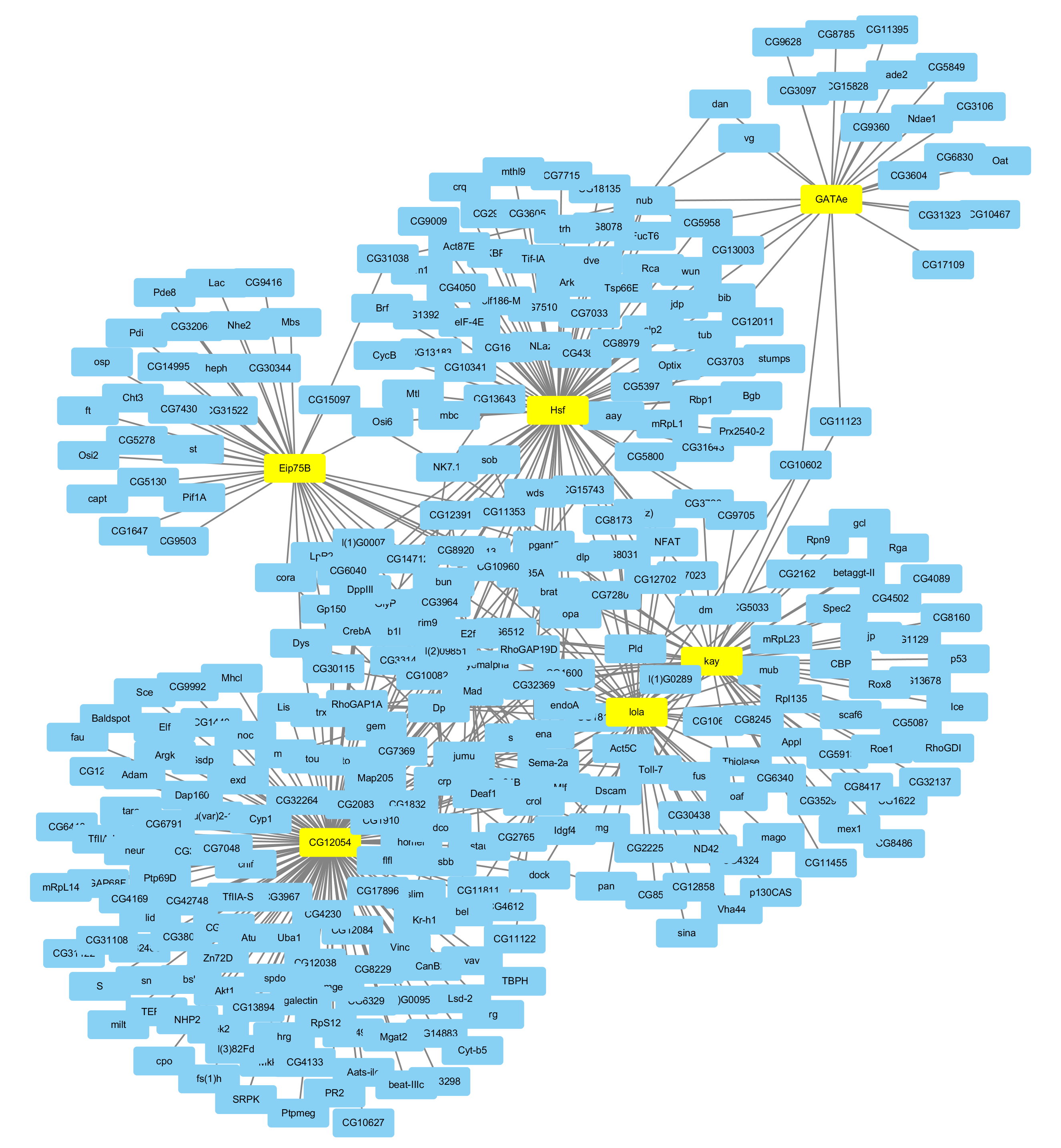

Supplement: S1 Fig — The six hub genes, i.e.CG12054, Hsf, kay, lola, Eip75B and GATAe, are highlighted with yellow background. (TIF) [file pcbi.1007324.s001.tif]

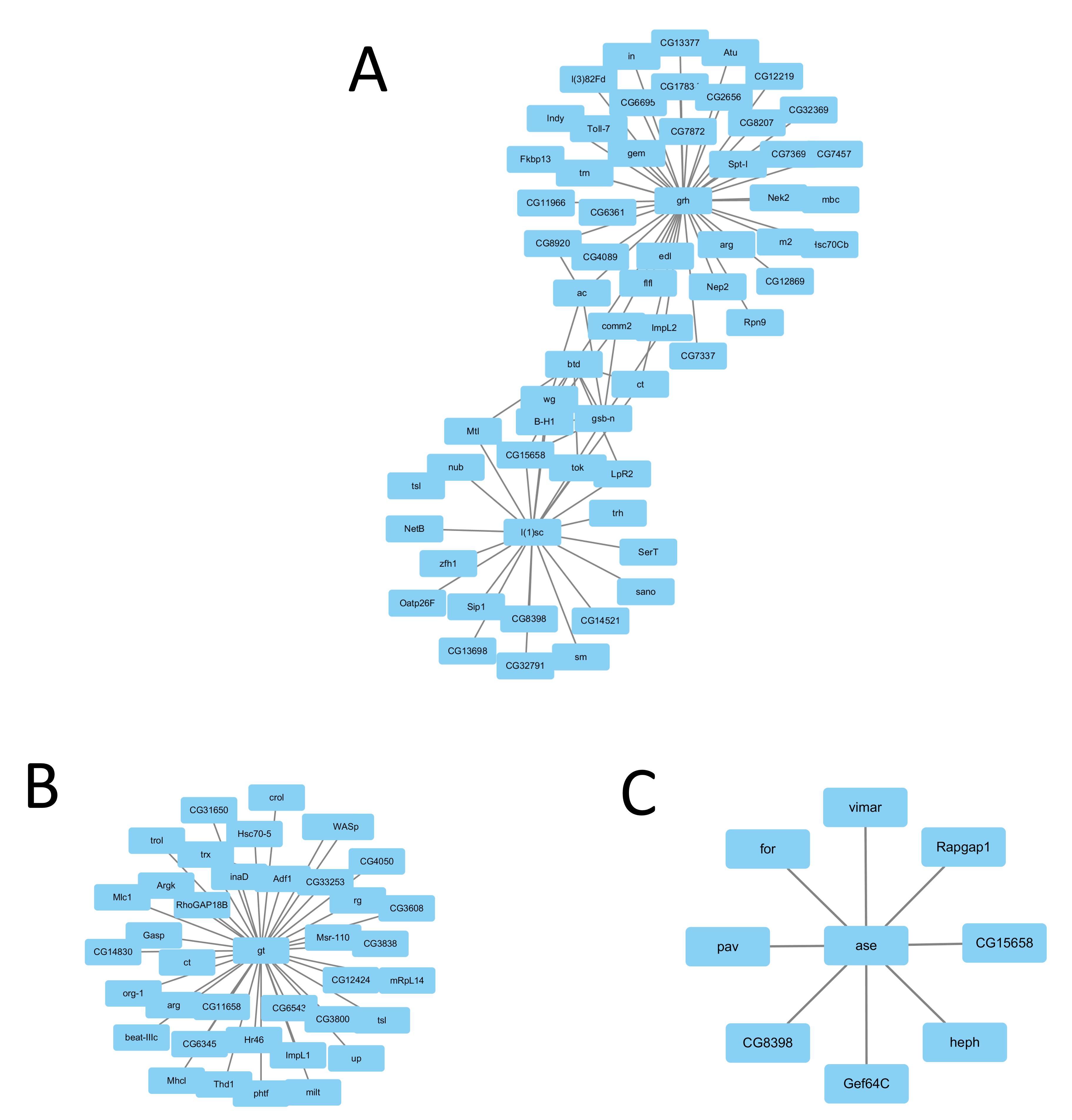

Supplement: S2 Fig — (A) shows the targets of grh and l(1)sc, (B) and (C) show the targets of gt and ase, respectively. (TIF) [file pcbi.1007324.s002.tif]
